# Effects of Dietary Supplementation With *Bacillus subtilis*, as an Alternative to Antibiotics, on Growth Performance, Serum Immunity, and Intestinal Health in Broiler Chickens

**DOI:** 10.3389/fnut.2021.786878

**Published:** 2021-11-29

**Authors:** Kai Qiu, Cheng-liang Li, Jing Wang, Guang-hai Qi, Jun Gao, Hai-jun Zhang, Shu-geng Wu

**Affiliations:** ^1^Risk Assessment Laboratory of Feed Derived Factors to Animal Product Quality Safety of Ministry of Agriculture and Rural Affairs, National Engineering Research Center of Biological Feed, Institute of Feed Research, Chinese Academy of Agricultural Sciences, Beijing, China; ^2^Animal Nutrition, Nutrition and Care, Evonik (China) Co., Ltd., Beijing, China

**Keywords:** *Bacillus subtilis*, broiler chickens, gut microbiota, immunity, probiotic

## Abstract

*Bacillus subtilis* (*B. subtilis*) as in-feed probiotics is a potential alternative for antibiotic growth promoters (AGP) in the poultry industry. The current study investigated the effects of *B. subtilis* on the performance, immunity, gut microbiota, and intestinal barrier function of broiler chickens. A 42-day feeding trial was conducted with a total of 600 1-day-old Arbor Acres broilers with similar initial body weight, which was randomly divided into one of five dietary treatments: the basal diet (Ctrl), Ctrl + virginiamycin (AGP), Ctrl + *B. subtilis* A (BSA), Ctrl + *B. subtilis* B (BSB), and Ctrl + *B. subtilis* A + B (1:1, BSAB). The results showed significantly increased average daily gain in a step-wise manner from the control, *B. subtilis*, and to the AGP groups. The mortality rate of the *B. subtilis* group was significantly lower than the AGP group. The concentrations of serum immunoglobulin (Ig) G (IgG), IgA, and IgM in the *B. subtilis* and AGP groups were higher than the control group, and the *B. subtilis* groups had the highest content of serum lysozyme and relative weight of thymus. Dietary *B. subtilis* increased the relative length of ileum and the relative weight of jejunum compared with the AGP group. The villus height (V), crypt depth (C), V/C, and intestinal wall thickness of the jejunum in the *B. subtilis* and AGP groups were increased relative to the control group. Dietary *B. subtilis* increased the messenger RNA (mRNA) expression of ZO-1, Occludin, and Claudin-1, the same as AGP. The contents of lactic acid, succinic acid, and butyric acid in the ileum and cecum were increased by dietary *B. subtilis*. Dietary *B. subtilis* significantly increased the *lactobacillus* and *bifidobacteria* in the ileum and cecum and decreased the *coliforms* and *Clostridium perfringens* in the cecum. The improved performance and decreased mortality rate observed in the feeding trial could be accrued to the positive effects of *B. subtilis* on the immune response capacity, gut health, and gut microflora balance, and the combination of two strains showed additional benefits on the intestinal morphology and tight junction protein expressions. Therefore, it can be concluded that dietary *B. subtilis* A and B could be used as alternatives to synthetic antibiotics in the promotion of gut health and productivity index in broiler production.

## Introduction

Antibiotics have been widely used as a growth promoter and also to enhance the immunocompetence of birds against infectious diseases (1). Currently, the global trend in animal production is toward a reduction or ban on the use of feed antibiotics for growth [antibiotic growth promoter (AGP)] and an increase in the application of non-antibiotic approaches that can provide similar benefits. This is accrued to the fact that the widespread use of antibiotics over 50 years has led to the emergence of resistant bacteria and drug residues in animal products (2–4). In the context of growing consumer preference for antibiotic-free meat products, researchers in livestock production and poultry sectors have focused on finding alternatives to replace synthetic antibiotics used in most ongoing therapeutic regimes. There is an interest to characterize probiotics as a kind of viable alternatives that can promote the growth and health status of poultry through multiple ways/mechanisms(5–7).

Despite the large amount of microorganisms serving as probiotics in poultry production, the form of supplemental probiotics through the hostile environment such as low pH value and high concentration of bile salt within the gastrointestinal tract is a severe challenge for their survival (8). As a result, spore-forming bacteria such as *Bacillus subtilis* (*B. subtilis*) are gaining interest in animal health-related functional additive research due to their high tolerance and survivability under hostile environments in the gastrointestinal tract (9–11). Obviously, *B. subtilis* when applied in feed does not lose its viability due to its high stability and extended shelf life, hence a comparative advantage (10). Supplemental *B. subtilis* in poultry diets has many beneficial claims, including immune-modulation, enhanced nutrient digestibility, along with improvements in gut health, immunity, and growth performance in animals (12–14). However, many properties of probiotic bacteria vary as a function of strain (15). With respect to *B. subtilis*, its probiotic effects are highly strain-specific, and the underlying mechanisms of action remain largely elusive (16). It has been reported that the effects of dietary *B. subtilis* supplementation on growth performance and intestinal physiology in broilers were markedly strain-dependent (17, 18), hence the need for continuous studies on the various strains of *B. subtilis* to understand their mechanisms of action in these animals.

*Bacillus subtilis* A and B were selected as two potential probiotic strains through a multi-parameter selection process for their ability to remain viable in feed and through the harsh conditions of the upper gastrointestinal tract. However, far less is known about the effects of these two strains on broilers and their potential roles to be AGP substitutes. Therefore, this study aimed to evaluate the individual or combined effect of two strains of *B. subtilis* A and B on the growth performance, serum immunity, gut microbiota, and intestinal barrier function of broiler chickens.

## Materials and Methods

### Experimental Design and Bird Management

A total of 600 newly hatched male Arbor Acres (AA) broiler chicks with an average body weight (BW) of 40.09 g were obtained from a local hatchery and assigned into five dietary treatments in a randomized complete block design with 10 replicates per treatment. Each replicate contained 12 chicks (half male and half female) housed in two cages with male and female apart. The 42-day trial spanned three phases including the starter period (day 0–14), grower period (day 15–28), and finisher period (day 29–42). Three basal diets (cold pellet form) were formulated according to the nutrient requirements of AMINOChick®2.0 and the Chinese Feeding Standard of Chicken (NY/T, 33-2018), and their ingredient composition and nutrient levels are shown in Table 1. The control group was fed basal diets. The AGP group was fed basal diets supplemented with 15 mg/kg virginiamycin. Three probiotics groups were fed basal diets supplemented with 500 mg/kg *B. subtilis* A (BSA) (2E9 CFU/g), 500 mg/kg *B. subtilis* B (BSB) (2E9 CFU/g), or 500 mg/kg mixture of BSA and BSB (1:1, named as BSAB), respectively. Diet samples collected from all the treatments and phases were sent to Evonik Operations GmbH for the proximate and spore count analysis. The pre-products are both generally recognized as safe (GRAS) under the Association of American Feed Control Officials (AAFCO) definition 36.14 and contain a guaranteed minimum of 2 × 10^9^ CFU/g.

**Table 1:** Ingredient and calculated nutrient compositions of basal diet.

**Items**	**Day 1–14**	**Day 15–28**	**Day 29–42**
**Ingredients, %**			
Corn	58.20	58.70	61.00
Soybean meal	34.85	33.02	30.85
Soybean oil	2.54	4.48	4.72
CaHPO_4_	2.27	2.00	1.82
Limestone	0.82	0.72	0.70
Salt	0.35	0.35	0.35
DL-Methionine	0.34	0.26	0.2
L-Lysine·HCl	0.31	0.15	0.04
Vitamin premix^a^	0.02	0.02	0.02
Mineral premix^b^	0.20	0.20	0.20
Choline chloride (50%)	0.10	0.10	0.10
Total	100.00	100.00	100.00
**Nutrient levels** ^**c**^			
AME (MJ/kg)	12.35	12.97	13.18
Crude protein, %	22.00	21.00	20.00
Calcium, %	1.00	1.00	0.90
Available phosphorus, %	0.50	0.45	0.40
Lysine, %	1.29	1.15	1.09
Methionine, %	0.55	0.52	0.48
Methionine + cystine, %	0.94	0.84	0.84
Threonine, %	0.82	0.77	0.69
Tryptophan, %	0.24	0.18	0.22

a *Vitamin premix provided the following per kg of diets: VA, 12.500 IU; VD_3_, 2.500 IU; VK_3_, 2.65 mg; VB_1_, 2 mg; VB_2_, 6 mg; VB_12_, 0.025 mg; VE, 30 IU; biotin 0.0325 mg; folic acid, 1.25 mg; pantothenic acid, 12 mg; nicotinic acid, 50 mg*.

b *Mineral premix provided the following per kg of diets: Cu 8 mg, Zn 75 mg, Fe 80 mg, Mn 100 mg, Se 0.15 mg, I 0.35 mg*.

c *Nutritient levels were calculated values*.

*AME, apparent metabolizable energy*.

All the birds were raised in wire floor cages (cage size, 110 × 100 × 55 cm) in a three-level battery under environmentally controlled room conditions in the Nankou CAAS experimental base (Beijing, China). All the management was in accordance with the AA broiler management guide. A continuous incandescent white light was provided for the first 3 days, and then a 23L:1D lighting regime was maintained throughout the rest feeding trial. The room temperature was maintained at 33°C for the first week and then reduced by 3°C per week until it reached 24°C. Fresh feed and water on a daily basis were available *ad libitum* through individual feeders and drinkers in each cage. The chicks were vaccinated with inactivated Newcastle disease vaccine on days 7 and 21 and inactivated infectious bursal disease vaccine on days 14 and 28. The vaccines were purchased from Shanghai Haili Biotechnology Co., LTD (Shanghai, China). During the trial, the mortality of birds was recorded daily, and the feed consumption of the corresponding replicate was adjusted with their body weight accordingly. The feed intake (FI), BW, and mortality of each replicate were recorded every 2 weeks. The average daily feed intake (ADFI), average daily gain (ADG), and feed conversion ratio (FCR) were calculated based on FI and BW.

### Data and Sample Collection

On days 28 and 42, one chick weighing close to the average weight of the replicate was selected for sample collection after 12 h fasting. About 5 ml of blood was collected from the wing vein using a vacutainer tube, kept in a slanting position for 30 min, and then centrifuged at 3,000 rpm/min for 15 min at 4°C. The obtained serum samples were stored in 2 ml plastic vials at −20°C, pending for ELISA analysis.

Subsequently, the selected birds were euthanized and then dissected under aseptic conditions. Immune organs including the spleen, thymus, and bursa of Fabricius of the birds were weighed and their relative weight was calculated as the ratio of organ weight (g) to BW (kg). The whole duodenum, jejunum, ileum, and ceca were moved free of the mesentery and immediately placed on ice for sampling. About 4 g of digesta sample from the ileum and cecum were collected and immediately snap-frozen in liquid nitrogen followed by storage in −80°C until the short-chain fatty acids (SCFA) and microbiota analysis. Then, the relative index of the intestinal length (length/BW × 100%, cm/g) and weight (weight/BW × 100%, g/g) of the duodenum, jejunum, ileum, and cecum were calculated, respectively. About 3 cm of tissues from the duodenum (medial portion), jejunum (medial portion posterior to the bile ducts and anterior to Meckel's diverticulum), and ileum (medial portion posterior to Meckel's diverticulum and anterior to the ileocecal junction) were cut off gently in duplicate, one fixed in 10% formalin for histomorphology, the other immediately snap-frozen in liquid nitrogen and stored in −80°C until mRNA extraction.

### Histology and Histomorphology Analysis of the Intestine

The fixed intestinal samples were dehydrated, embedded in paraffin wax, cut into serial 5 μm sections, and stained by hematoxylin and eosin. Histological sections were examined by a microscope coupled with a Microcomp integrated digital imaging analysis system (Nikon Eclipse 80i, Nikon Co., Tokyo, Japan). Three orientated sections cutting vertically from the villus enterocytes to the muscularis mucosa were selected from each sample and the measurements were carried out as follows (19). The vertical distance from the villus tip to villus–crypt junction level was taken as the intestinal villus height (VH), and the vertical distance from the villus-crypt junction to the lower limit of the crypt as the crypt depth (CD). Ten loci per section were selected for the measurement of the VH, CD, and intestinal wall thickness (IWT). The ratio of VH/CD was calculated as V/C.

### RNA Extraction and Quantitative Real Time-PCR

The total DNA of the microbe from the intestinal digesta and the total RNA from the intestinal tissues were extracted using TRIZOL reagent (Invitrogen, Carlsbad, CA, USA) according to the instructions of the manufacturer. Samples of DNA and RNA were determined for integrity by 1% agarose gel electrophoresis and for concentration and purity using a NanoDrop ND-1000 spectrophotometer (Thermo Fisher Scientific, DE, USA). Then the RNA samples were treated with DNase I (Cwbio IT Group, Beijing, China) and converted into complementary DNA (cDNA) using a reverse transcription kit (Vazyme Biotech, Nanjing, China). An iCycler iQ5 multicolor RT-PCR detection system (Bio-Rad Laboratories, Hercules, CA, USA) and a RealMasterMix-SYBR Green kit [ChamQ SYBR Color qPCR Master Mix (2 ×), Vazyme, Nanjing] were used for the determination of gene expression according to the instructions of the manufacturer. The primers used in this study are listed in Table 2. The thermal cycling conditions of qRT-PCR were as follows: 95°C for 5 min; 40 cycles of 95°C for 10 s, 60°C for 30 s. The results of the absolute qRT-PCR of the microbe DNA were expressed as copies/g. The relative gene expression of the tissue samples was calculated using the 2^−ΔΔCt^ method.

**Table 2:** Primers used for quantitative reverse transcription PCR.

**Gene**	**Primer Sequences (5′-3′)**	**AT (°C)**	**Product size (bp)**
**Bacteria**			
*Lactobacillus*	F: AGCAGTAGGGAATCTTCCA	62	336
	R: CACCGCTACACATGGAG		
*Bifidobacteria*	F: TCGCGTC(C/T)GGTGTGAAAG	61	299
	R: CCACATCCAGC(A/G)TCCAC		
*Coliforms*	F: GTTAATACCTTTGCTCATTGA	62	341
	R: ACCAGGGTATCTTAATCCTGTT		
*Clostridium perfringens*	F:TGCACTATTTTGGAGATATAGATAC	60	287
	R:CTGCTGTGTTTATTTTATACTGTTC		
**Tight junction genes**			
*ZO-1*	F: CTTCAGGTGTTTCTCTTCCTCCTC	59	131
	R: CTGTGGTTTCATGGCTGGATC		
*ZO-2*	F: CGGCAGCTATCAGACCACTC	58	87
	R: CACAGACCAGCAAGCCTACAG		
*Occludin*	F: ACGGCAGCACCTACCTCAA	59	123
	R: GGGCGAAGAAGCAGATGAG		
*Claudin-1*	F:CATACTCCTGGGTCTGGTTGGT	59	100
	R:GACAGCCATCCGCATCTTCT		
*Claudin-5*	F: CATCACTTCTCCTTCGTCAGC	59	111
	R: GCACAAAGATCTCCCAGGTC		
*β-actin*	F:GAGAAATTGTGCGTGACATCA	60	152
	R: CCTGAACCTCTCATTGCCA		

*AT, annealing temperature*.

### Chemical Analysis

The serum lysozyme activity was measured using *Micrococcus lysodeikticus* cells as a substrate. The serum immunoglobulin (Ig) A (IgA), IgG, and IgM were analyzed by colorimetric method using commercial kits (H108, H106, H109, Nanjing Jiancheng Bioengineering Institute, Nanjing, China) according to the instructions of the manufacturer. The concentrations of lactic acid, succinic acid, and SCFA (formic acid, acetic acid, propionic acid, butyric acid, iso-butyric acid, valeric acid, isovaleric acid) in the ileal and cecal digesta were measured as previously described (20) using the Dionex ICS-3000 Ion Chromatography System (ThermoFisher Scientific Inc., Waltham, MA, USA).

### Statistical Analysis

The data of the *B. subtilis* group were generated from the data of the three groups, BSA, BAB, and BSAB, where six replicates in each treatment were averagely divided as two replicates of the *B. subtilis* group. All the data were subjected to a one-way ANOVA procedure for a completely randomized design using the General Linear Mode (GLM) procedures of SAS 9.2 (SAS Inst. Inc., Cary, NC, USA). The differences among the treatments were separated by Duncan's multiple range tests. *P* ≤ 0.05 was considered significant.

## Results

### Growth Performance

The growth performance of broiler chickens is shown in Table 3. On days 14 and 28, the BW of the birds fed with AGP was significantly higher (*P* ≤ 0.05) than those fed with the control and *B. subtilis* diets. At the end of the trial, the birds in the AGP group showed higher (*P* ≤ 0.05) BW than the *B. subtilis* groups whose BW was higher (*P* ≤ 0.05) than the control group. During the trial, the BW of the broiler chickens was similar among the three *B. subtilis* treatments on days 14, 28, and 42. During the starter phase (day 1–14), the broiler chickens in the AGP group showed increased (*P* ≤ 0.05) ADG, ADFI, and mortality rate and decreased FCR relative to the *B. subtilis* group which is not different from the control group, and all of these indexes were similar among the three *B. subtilis* groups. During the grower phase (day 15–28), the ADG and ADFI of the AGP and *B. subtilis* groups were significantly higher (*P* ≤ 0.05) than the control group. Among the *B. subtilis* groups, the BSB group showed higher (*P* ≤ 0.05) FCR than the BSAB group, and both of them did not vary with that of the BSA group. In the finisher phase (day 29–42), the ADG of the broiler chickens in the AGP group was higher (*P* ≤ 0.05) than those in the *B. subtilis* and control groups. The FCR of the AGP group was decreased (*P* ≤ 0.05) as compared with the control and *B. subtilis* groups. No differences in ADG, ADFI, FCR, and mortality rate were observed among the three *B. subtilis* groups. During the whole trial period, day 1–42, the ADG of the broiler chickens showed a significant increase (*P* ≤ 0.05) in a stepwise manner from the control, *B. subtilis*, to the AGP groups. The birds in the *B. subtilis* group had higher (*P* ≤ 0.05) ADFI than the control group. The FCR of the AGP group was improved (*P* ≤ 0.05) relative to the control and *B. subtilis* groups. The mortality rate of the *B. subtilis* group was significantly (*P* ≤ 0.05) decreased as compared with the AGP group. There was no variation in growth performance indices among the broilers fed with diets supplemented with the three *B. subtilis* groups during the entire feeding phase.

**Table 3:** Effects of dietary *Bacillus subtilis* on growth performance of broiler chickens.

**Items** ^**1**^	**ADD**			**BS**			**SEM**	***p*** **-value**	
	**Ctrl**	**AGP**	**BS**	**BSA**	**BSB**	**BSAB**		**ADD**	**BS**
**BW, g**									
Day 0	40.09	40.09	40.09	40.09	40.09	40.09	0.00	1.00	1.00
Day 14	484.5^b^	510.5^a^	478.9^b^	475.8	477.9	482.8	6.20	<0.01	0.79
Day 28	1,379.3^b^	1,458.5^a^	1,416.4^b^	1,404.6	1,398.5	1,446.0	17.52	0.01	0.19
Day 42	2,403.6^c^	2,564.4^a^	2,474.6^b^	2,440.6	2,454.5	2,528.6	29.23	<0.01	0.16
**Day 1–14**									
ADG, g	31.74^b^	33.60^a^	31.34^b^	31.12	31.27	31.62	0.44	<0.01	0.79
ADFI, g	35.40^ab^	36.13^a^	34.99^b^	34.75	34.9	35.33	0.50	0.15	0.74
FCR, g/g	1.12^a^	1.07^b^	1.12^a^	1.12	1.12	1.12	0.01	<0.01	1.00
Mortality, %	0.00^b^	1.67^a^	0.00^b^	0.00	0.00	0.00	0.50	0.01	1.00
**Day 15–28**									
ADG, g	63.92^b^	67.72^a^	66.97^a^	66.34	65.76	68.8	1.07	0.03	0.16
ADFI, g	96.75^b^	101.88^a^	102.99^a^	102.64	103.38	102.94	1.52	<0.01	0.96
FCR, g/g	1.52	1.51	1.54	1.55^xy^	1.57^x^	1.50^y^	0.02	0.32	0.06
Mortality, %	0.00	0.83	0.55	0.83	0.00	0.83	0.65	0.64	0.61
**Day 29–42**									
ADG, g	73.16^b^	78.99^a^	75.59^b^	74	75.43	77.33	1.45	0.02	0.36
ADFI, g	144.88	147.63	148.22	146.25	146.19	152.21	2.21	0.45	0.17
FCR, g/g	1.98^a^	1.87^b^	1.96^a^	1.98	1.94	1.97	0.03	0.01	0.59
Mortality, %	0.00	1.91	0.91	0.00	1.82	0.91	0.89	0.32	0.35
**Day 1–42**									
ADG, g	56.27^c^	60.10^a^	57.97^b^	57.16	57.49	59.25	0.70	<0.01	0.16
ADFI, g	92.34^b^	95.21^ab^	95.40^a^	94.55	94.82	96.83	1.17	0.08	0.47
FCR, g/g	1.64^a^	1.56^b^	1.64^a^	1.65	1.65	1.63	0.01	<0.01	0.50
Mortality, %	0.00^b^	4.17^a^	1.39^b^	0.83	1.67	1.67	1.15	0.04	0.80

*^a, b, c, x, y^Mean within a row in ADD or BS with no common superscripts differ significantly (P < 0.05)*.

1 *Data were the mean of 10 replicates with 12 birds each*.

*BW, body weight; ADG, average daily gain; ADFI, average daily feed intake; FCR, feed conversion ratio (feed intake/weight gain, g:g); ADD, additives; Ctrl, the control group; AGP, antibiotic growth promoter; BS, B. subtilis; BSA, B. subtilis A; BSB, B. subtilis B; BSAB, the mixture of BSA and BSB (1:1)*.

### The Relative Weight of Immune Organ and Serum Immunity

**Figure 1:**
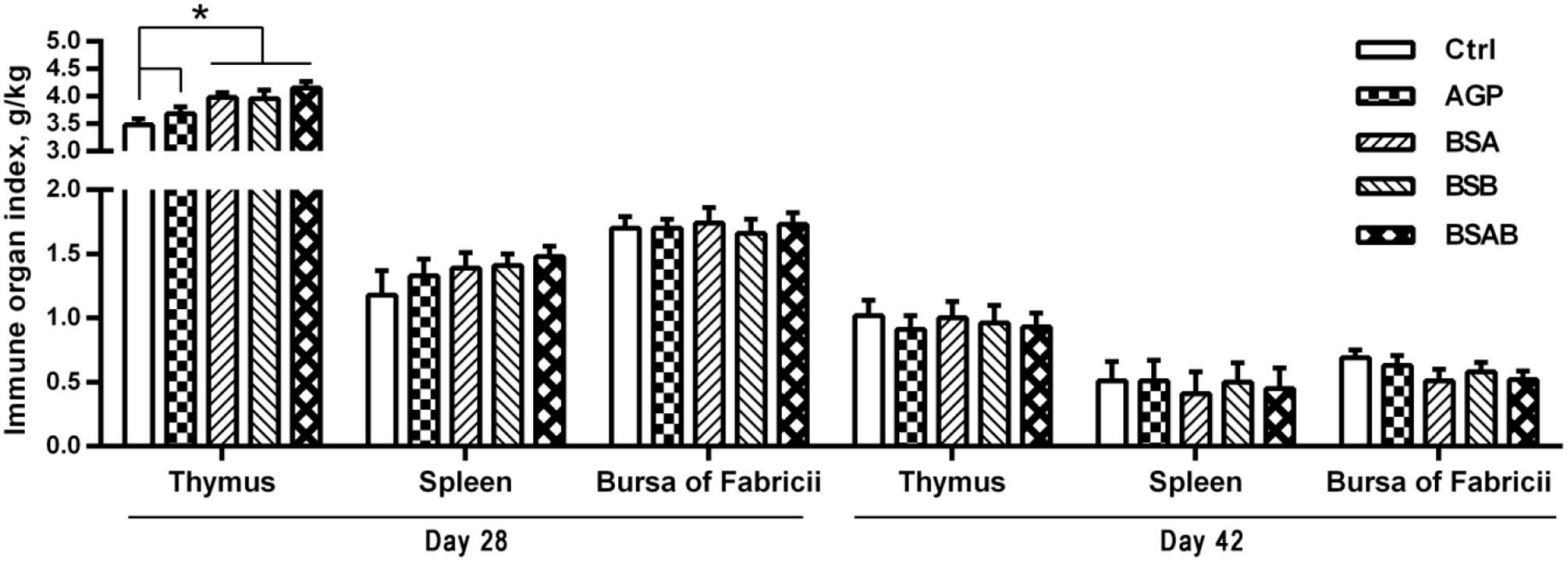
Effects of dietary *Bacillus subtilis* on the immune organ index of broiler chickens (*n* = 10, g/kg). ADD, additives; Ctrl, the control group; AGP, antibiotic growth promoter; BS, *B. subtilis*; BSA, *B. subtilis* A; BSB, *B. subtilis* B; BSAB, the mixture of BSA and BSB (1:1). *Means significant different (*P* < 0.05).

As shown in Figure 1, at day 28, all the three *B. subtilis* groups had higher (*P* ≤ 0.05) relative weight of thymus compared with the control and AGP groups. On day 42, the relative weights of the immune organs including the thymus, spleen, and bursa of fabricius were not influenced by the dietary treatments. The effects of the dietary treatments on the concentration of IgA, IgG, IgM, and lysozyme in the serum are shown in Table 4. On day 28, the level of serum IgG and IgA was higher (*P* ≤ 0.05) in the AGP and *B. subtilis* groups as compared with the control group, while no differences were observed among the *B. subtilis* groups. On day 42, the concentration of serum IgA in the *B. subtilis* and AGP groups was higher (*P* ≤ 0.05) compared with the control group. The *B. subtilis* group showed a higher (*P* ≤ 0.05) content of serum lysozyme than the control and AGP groups. The contents of serum IgA, IgG, and IgM did not differ among the three *B. subtilis* groups at day 42, but the concentration of serum lysozyme was higher (*P* ≤ 0.05) in the BSAB group than in the BSB group.

**Table 4:** Effects of dietary *B. subtilis* on serum immunity of broiler chickens.

**Items** ^**1**^	**ADD**			**BS**			**SEM**	***p*** **-value**	
	**Ctrl**	**AGP**	**BS**	**BSA**	**BSB**	**BSAB**		**ADD**	**BS**
**Day 28**									
IgG, g/L	4.15^b^	4.26^a^	4.23^a^	4.26	4.21	4.23	0.03	0.07	0.59
IgA, g/L	2.14^b^	2.27^a^	2.22^a^	2.22	2.20	2.25	0.03	<0.01	0.54
IgM, g/L	1.60^b^	1.65^ab^	1.66^a^	1.65	1.66	1.68	0.02	0.02	0.64
Lysozyme, mg/L	4.65^b^	4.33^b^	5.34^a^	5.07	5.36	5.59	0.29	<0.01	0.31
**Day 42**									
IgG, g/L	4.14	4.16	4.21	4.18	4.21	4.24	0.04	0.20	0.50
IgA, g/L	2.19^b^	2.29^a^	2.26^a^	2.24	2.25	2.30	0.03	0.03	0.33
IgM, g/L	1.64	1.65	1.66	1.65	1.67	1.67	0.02	0.54	0.67
Lysozyme, mg/L	3.97^b^	3.85^b^	4.75^a^	4.71^xy^	4.48^y^	5.07^x^	0.21	<0.01	0.10

*^a, b, x, y^Mean within a row in ADD or BS with no common superscripts differ significantly (P < 0.05)*.

1 *Data are the mean of 10 replicates*.

*ADD, additives; Ctrl, the control group; AGP, antibiotic growth promoter; BS, B. subtilis; BSA, B. subtilis A; BSB, B. subtilis B; BSAB, the mixture of BSA and BSB (1:1)*.

### Intestinal Development

The relative length and weight of the duodenum, jejunum, ileum, and cecum are presented in Table 5. On day 28, the relative length of the duodenum and ileum of the *B. subtilis* group was longer (*P* ≤ 0.05) than those of the AGP group, while similar to the control group. The *B. subtilis* group showed increased (*P* ≤ 0.05) relative length of the cecum as compared with the control group. On day 42, the relative length of the ileum was significantly longer (*P* ≤ 0.05) in the *B. subtilis* and control groups than in the AGP group. As for the relative weight of the duodenum, jejunum, ileum, and cecum at days 28 and 42, only the relative weight of the jejunum in the *B. subtilis* group was greater (*P* ≤ 0.05) than the AGP group at day 28. The relative length and weight of the duodenum, jejunum, ileum, and cecum at days 28 and 42 did not differ between the three *B. subtilis* groups.

**Table 5:** Effects of dietary *B. subtilis* on the intestinal index in broiler chickens.

**Items** ^**1**^	**ADD**			**BS**			**SEM**	***p*** **-value**	
	**Ctrl**	**AGP**	**BS**	**BSA**	**BSB**	**BSAB**		**ADD**	**BS**
**Relative length, cm/kg BW**									
**Day 28**									
Duodenum	9.89^ab^	9.54^b^	10.22^a^	10.21	10.17	10.28	0.27	0.09	0.94
Jejunum	56.07	54.10	56.24	56.94	55.84	55.95	1.35	0.37	0.83
Ileum	55.32^a^	51.71^b^	56.02^a^	56.09	54.95	57.02	1.23	0.01	0.47
Cecum	4.11^b^	4.41^ab^	4.66^a^	4.68	4.75	4.54	0.17	0.03	0.64
**Day 42**									
Duodenum	5.96	5.99	6.01	6.07	5.87	6.09	0.19	0.97	0.65
Jejunum	33.61	31.60	32.40	32.33	31.23	33.63	1.08	0.42	0.31
Ileum	32.78^a^	29.81^b^	33.47^a^	32.78	33.04	34.59	1.04	0.01	0.41
Cecum	8.34	8.16	8.58	8.72	8.33	8.68	0.25	0.30	0.46
**Relative weight, g/kg BW**									
**Day 28**									
Duodenum	10.11	10.16	10.75	10.89	10.72	10.64	0.47	0.35	0.92
Jejunum	16.11^ab^	15.08^b^	16.94^a^	16.91	16.70	17.21	0.52	<0.01	0.78
Ileum	13.74	13.38	14.24	14.18	13.99	14.56	0.61	0.44	0.76
Cecum	3.40	3.54	3.73	3.71	3.58	3.90	0.16	0.18	0.29
**Day 42**									
Duodenum	7.32	7.58	7.60	7.57	7.77	7.45	0.35	0.78	0.80
Jejunum	12.05	11.72	12.17	12.29	11.90	12.33	0.47	0.70	0.77
Ileum	11.00	10.49	10.33	10.64	10.13	10.21	0.44	0.43	0.69
Cecum	4.64	4.54	4.40	4.58	4.21	4.40	0.34	0.81	0.66

*^a, b^Means within a row in ADD with no common superscripts differ significantly (P < 0.05)*.

1 *Data were the mean of 10 replicates*.

*BW, body weight; ADD, additives; Ctrl, the control group; AGP, antibiotic growth promoter; BS, B. subtilis; BSA, B. subtilis A, named as Gutcare; BSB, B. subtilis B, named as Gutplus; BSAB, the mixture of BSA and BSB (1:1)*.

The results of the intestinal morphology are shown in Table 6. With respect to the duodenum, at day 28, the VH, V/C, and IWT were higher (*P* ≤ 0.05) in the *B. subtilis* group than in the control group. The CD of the AGP group increased (*P* ≤ 0.05) relative to the control group, while both of them were similar to the *B. subtilis* group. The CD of the BSB group was significantly deeper (*P* ≤ 0.05) than the BSA and BSAB groups. On day 42, the IWT of the AGP group was thicker (*P* ≤ 0.05) than the control group, while both of them were not different from the *B. subtilis* group. The VH, CD, V/C, and IWT did not differ between the three *B. subtilis* groups. As for jejunum, at day 28, the *B. subtilis* group showed increased (*P* ≤ 0.05) VH, V/C, and IWT relative to the control group, while it was similar with the AGP group. No differences in intestinal morphology were found between the three *B. subtilis* groups. On day 42, the CD and IWT of the *B. subtilis* and AGP groups were increased (*P* ≤ 0.05) relative to the control group. The VH and IWT of the BSAB group were higher (*P* ≤ 0.05) than the BSA and BSB groups. The CD of the BSB group was deeper (*P* ≤ 0.05) than the control group, but not different from the BSAB group. The V/C of the BSAB and control groups was bigger (*P* ≤ 0.05) than the BSB group. Regarding the ileum, no differences between the control, AGP, and *B. subtilis* groups were found for VH, CD, V/C, and IWT at days 28 and 42. The V/C of the BSAB group was greater (*P* ≤ 0.05) than the BSA group on day 28, and both of them were not significantly different from the BSB group. On day 42, the BSA and BSAB groups showed bigger (*P* ≤ 0.05) VH and V/C than the BSB group. The IWT of the BSAB group was thicker (*P* ≤ 0.05) than the BSB group.

**Table 6:** Effects of dietary *B. subtilis* on the intestinal morphology of broiler chickens.

**Items** ^**1**^	**ADD**			**BS**			**SEM**	***p-*** **Value**	
	**Ctrl**	**AGP**	**BS**	**BSA**	**BSB**	**BSAB**		**ADD**	**BS**
**Duodenum**									
**Day 28**									
Villus height, μm	1,357.91^b^	1,473.48^ab^	1,532.00^a^	1,494.21	1,553.94	1,547.86	28.05	0.05	0.74
Crypt depth, μm	361.03^a^	315.18^b^	340.75^ab^	320.88^y^	380.00^x^	321.38^y^	6.64	0.09	<0.01
V/C	3.76^b^	4.72^a^	4.57^a^	4.68	4.16	4.86	0.12	0.01	0.18
Intestinal wall thickness, μm	1,931.55^b^	2,016.86^ab^	2,097.42^a^	2,066.56	2,106.48	2,119.21	0.09	0.06	0.82
**Day 42**									
Villus height, μm	1,630.94	1,784.70	1,716.55	1,766.66	1,646.54	1,736.44	33.62	0.36	0.46
Crypt depth, μm	390.30	400.70	375.31	390.05	373.63	362.25	7.29	0.37	0.43
V/C	4.22	4.53	4.63	4.58	4.47	4.82	0.12	0.42	0.60
Intestinal wall thickness, μm	2,362.98^b^	2,596.56^a^	2,469.63^ab^	2,532.55	2,384.14	2,492.20	36.78	0.13	0.36
**Jejunum**									
**Day 28**									
Villus height, μm	962.50^b^	1,113.40^a^	1,177.42^a^	1,115.36	1,193.50	1,223.39	23.41	<0.01	0.27
Crypt depth, μm	260.60	283.20	286.15	275.89	289.53	293.03	6.29	0.29	0.62
V/C	3.76^b^	3.98^ab^	4.14^a^	4.05	4.18	4.19	0.07	0.13	0.78
Intestinal wall thickness, μm	1,388.21^b^	1,640.52^a^	1,717.33^a^	1,627.51	1,752.99	1,771.50	73.82	<0.01	0.27
**Day 42**									
Villus height, μm	1,343.50	1,535.00	1,557.92	1,507.43^y^	1,408.04^y^	1,758.29^x^	46.73	0.20	0.02
Crypt depth, μm	295.61^b^	346.66^a^	373.53^a^	351.54^y^	401.03^x^	368.03^xy^	8.64	<0.01	0.10
V/C	4.54	4.44	4.21	4.34^x^	3.51^y^	4.77^x^	0.12	0.50	<0.01
Intestinal wall thickness, μm	1,922.39^b^	2,302.98^a^	2,308.46^a^	2,207.90^y^	2,201.85^y^	2,515.64^x^	59.15	0.03	0.07
**Ileum**									
**Day 28**									
Villus height, μm	700.84	742.28	708.80	687.63	702.75	736.01	16.12	0.68	0.65
Crypt depth, μm	219.64	248.53	226.55	230.84	231.79	217.04	6.03	0.27	0.59
V/C	3.23	3.07	3.15	2.98^y^	3.04^xy^	3.43^x^	0.07	0.78	0.07
Intestinal wall thickness, μm	1,162.04	1,272.2	1,123.51	1,032.69	1,157.81	1,180.04	18.98	0.21	0.27
**Day 42**									
Villus height, μm	834.34	1,069.24	1,036.99	1,114.95^x^	771.98^y^	1,224.05^x^	48.07	0.21	0.02
Crypt depth, μm	222.26	259.4	251.98	243.56	249.53	262.86	8.04	0.28	0.72
V/C	3.81	4.17	4.11	4.46^x^	3.10^y^	4.77^x^	0.15	0.72	0.01
Intestinal wall thickness, μm	1,396.92	1,720.41	1,621.81	1,618.35^xy^	1,388.10^y^	1,858.98^x^	59.32	0.20	0.07

*^a, b, x, y^Mean within a row in ADD or BS with no common superscripts differ significantly (P < 0.05)*.

1 *Data are the mean of 10 replicates*.

*ADD, additives; Ctrl, the control group; AGP, antibiotic growth promoter; BS, B. subtilis; BSA, B. subtilis A; BSB, B. subtilis B; BSAB, the mixture of BSA and BSB (1:1); V/C, the ratio of Villus height to Crypt depth*.

**Figure 2:**
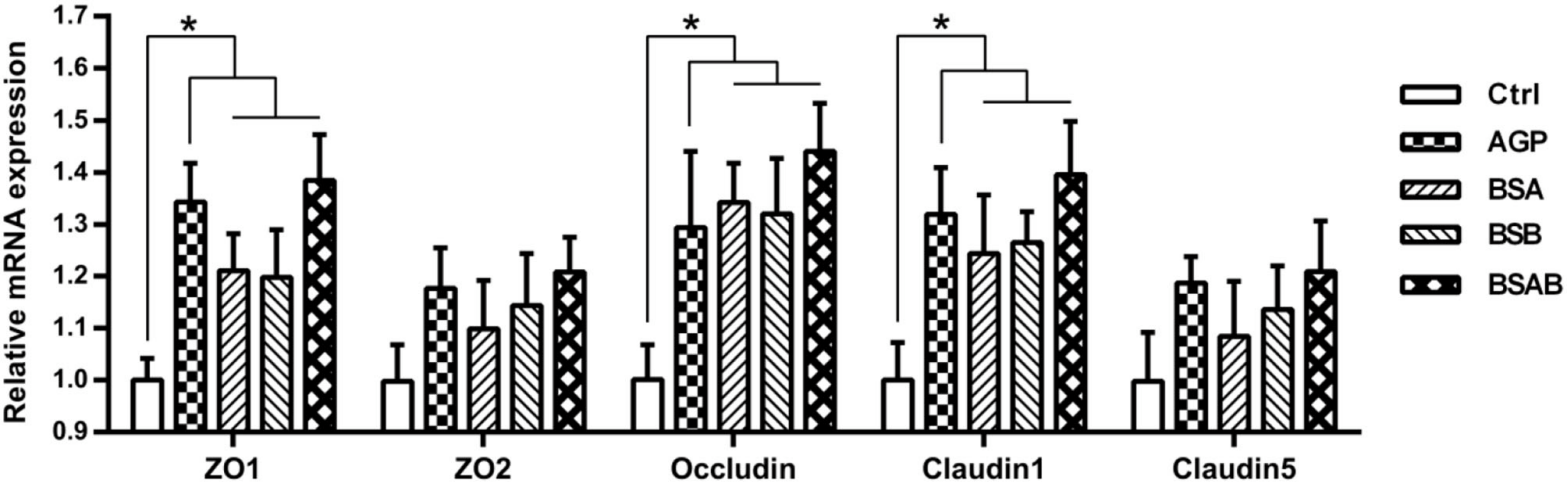
Effects of dietary *B. subtilis* on intestinal tight junction protein genes expressions in the jejunum of broiler chickens (*n* = 10). ADD, additives; Ctrl, the control group; AGP, antibiotic growth promoter; BS, *B. subtilis*; BSA, *B. subtilis* A; BSB, *B. subtilis* B; BSAB, the mixture of BSA and BSB (1:1). *Means significant difference between the groups (*P* < 0.05).

The mRNA expressions of the genes encoding intestinal tight junction proteins in the jejunum including ZO1, ZO2, Occludin, Claudin 1, and Claudin 5 are shown in Figure 2. There were no differences between the three *B. subtilis* groups for these gene expressions. The *B. subtilis* and AGP groups showed higher (*P* ≤ 0.05) mRNA expression of ZO-1, Occludin, Claudin-1 than the control group.

### Composition of Organic Acid and Microbiota in Digesta

As shown in Table 7, the concentration of lactic acid and succinic acid in the ileal digesta of the *B. subtilis* group was higher (*P* ≤ 0.05) than the control and AGP groups. The *B. subtilis* group was found to have higher (*P* ≤ 0.05) content of propionic acid and butyric acid in the ileal digesta than the AGP group, while it is not different from the control group. The content of formic acid, acetic acid, and total SCFA did not differ between the control, AGP, and *B. subtilis* groups. Except that the BSB group showed higher (*P* ≤ 0.05) content of total SCFA than the BSA group, the concentration of other organic acids in the ileal digesta was similar between the three *B. subtilis* groups. With regard to the cecum, the concentration of lactic acid, succinic acid, formic acid, butyric acid, and isovaleric acid in the digesta of the *B. subtilis* group was higher (*P* ≤ 0.05) than the control and AGP groups. The *B. subtilis* group showed higher (*P* ≤ 0.05) content of isobutyric acid in the digesta than the AGP group, but similar with the control group. There are no differences in organic acids content in the digesta among the three *B. subtilis* groups.

**Table 7:** Effects of dietary *B. subtilis* on short-chain fatty acid content in the ileum and cecum digesta of broiler chickens.

**Digesta**^**1**^, **mg/kg**	**ADD**			**BS**			**SEM**	***p*** **-value**	
	**Ctrl**	**AGP**	**BS**	**BSA**	**BSB**	**BSAB**		**ADD**	**BS**
**Ileum**									
Lactic acid	4,700^b^	4,514^b^	5,773^a^	5,528	5,933	5,858	435	0.02	0.81
Succinic acid	26.59^b^	29.89^b^	40.58^a^	38.09	41.20	42.46	4.42	0.01	0.81
Total SCFA	319.3	310.4	332.7	263.6^y^	407.5^x^	326.8^xy^	43.9	0.83	0.12
Formic acid	21.79	25.41	24.72	21.47	26.89	25.79	3.14	0.64	0.55
Acetic acid	286.9	269.6	311.1	322.2	321.7	289.3	46.1	0.69	0.88
Propionic acid	6.45^ab^	5.58^b^	7.62^a^	6.96	7.05	8.86	0.85	0.11	0.17
Butyric acid	4.15^ab^	3.41^b^	4.71^a^	4.46	4.41	5.26	0.39	0.02	0.17
**Cecum**									
Lactic acid	3.88^b^	3.75^b^	5.21^a^	5.13	4.88	5.63	0.48	0.01	0.59
Succinic acid	65.58^b^	79.10^b^	107.66^a^	95.30	113.96	113.71	11.14	<0.01	0.52
Total SCFA	6,218	6,437	6,779	6,668	6,667	7,001	416	0.45	0.80
Formic acid	29.94^b^	32.01^b^	43.96^a^	39.31	44.64	47.93	4.04	<0.01	0.41
Acetic acid	3,725	3,892	3,952	3,887	3,893	4,075	326	0.83	0.90
Propionic acid	1,597	1,659	1,732	1,723	1,682	1,790	145	0.69	0.82
Butyric acid	617.1^b^	603.3^b^	742.8^a^	721.4	741.0	765.9	40.2	<0.01	0.71
Isobutyric acid	87.14^ab^	79.30^b^	99.02^a^	96.69	95.83	104.53	7.67	0.07	0.62
Valeric acid	94.0	104.9	108.8	106.7	112.1	107.7	10.3	0.44	0.94
Isovaleric acid	67.69^b^	66.21^b^	97.16^a^	93.80	98.23	99.45	7.14	<0.01	0.73

*^a, b, x, y^Mean within a row in ADD or BS with no common superscripts differ significantly (P < 0.05)*.

1 *Data were the mean of 10 replicates*.

*ADD, additives; Ctrl, the control group; AGP, antibiotic growth promoter; BS, B. subtilis; BSA, B. subtilis A, named as Gutcare; BSB, B. subtilis B, named as Gutplus; BSAB, the mixture of BSA and BSB (1:1)*.

The effects of experimental treatments on intestinal microflora including *Lactobacillus, Bifidobacteria, Coliforms*, and *Clostridium perfringens* are presented in Table 8. For the ileum microflora, the amount of *Lactobacillus* in the *B. subtilis* group was higher (*P* ≤ 0.05) than the control and AGP groups, and the amount of *Bifidobacteria* in the *B. subtilis* group was higher (*P* ≤ 0.05) than the AGP group, and similar with the control group. No differences existed between the three *B. subtilis* groups about the content of microflora. For the cecum microflora, the amount of *Lactobacillus* and *Bifidobacteria* in the *B. subtilis* group was higher (*P* ≤ 0.05) than the AGP group, but no difference was found in the control group. The amounts of *coliforms* and *Clostridium perfringens* in the AGP and *B. subtilis* group were increased (*P* ≤ 0.05) than the control group. The number of *coliforms* in the BSAB group was higher (*P* ≤ 0.05) than the BSA group, and did not differ from the BSB group.

**Table 8:** Effects of dietary *B. subtilis* on ileum and cecum microbial populations of broiler chickens.

**Items**^**1**^, **copies/g**	**ADD**			**BS**			**SEM**	***p*** **-value**	
	**Ctrl**	**AGP**	**BS**	**BSA**	**BSB**	**BSAB**		**ADD**	**BS**
**Ileum**									
*Lactobacillus* (×10^9^)	2.44^b^	2.03^b^	3.22^a^	3.08	3.10	3.49	0.30	<0.01	0.63
*Bifidobacteria* (×10^6^)	0.39^ab^	0.29^b^	0.45^a^	0.42	0.47	0.47	0.06	0.04	0.79
*Coliforms* (×10^7^)	2.86	2.19	2.55	2.63	2.35	2.68	0.29	0.27	0.67
*Clostridium perfringens* (×10^5^)	4.12	3.58	3.76	3.66	3.86	3.76	0.38	0.58	0.94
**Cecum**									
*Lactobacillus* (×10^9^)	4.03^ab^	3.25^b^	4.78^a^	4.56	4.50	5.27	0.46	0.02	0.34
*Bifidobacteria* (×10^6^)	3.78^ab^	2.56^b^	4.68^a^	4.26	4.41	5.37	0.54	<0.01	0.13
*Coliforms* (×10^7^)	5.29^a^	2.86^b^	3.80^b^	4.40^x^	3.95^xy^	3.04^y^	0.46	<0.01	0.82
*Clostridium perfringens* (×10^5^)	7.51^a^	5.14^b^	5.47^b^	5.48	5.73	5.19	0.60	<0.01	0.49

*^a, b, x, y^Mean within a row in ADD or BS with no common superscripts differ significantly (P < 0.05)*.

1 *Data were the mean of 10 replicates*.

*ADD, additives; Ctrl, the control group; AGP, antibiotic growth promoter; BS, B. subtilis; BSA, B. subtilis A; BSB, B. subtilis B; BSAB, the mixture of BSA and BSB (1:1)*.

## Discussion

Over the past decades, therapeutic regimes in animal production globally entail about 70% use of synthetic antibiotics, probably due to their ability to improve gut health, hence health status and production index. However, the wide use of antibiotics as growth promoters in food animals has raised a lot of public health concerns (21). Along with the increasing consumer awareness of antimicrobial resistance and food safety, the use of AGP in animal agriculture was successively banned or restricted by the European Union, South Korea, the United States, and China. Such a scenario may reduce the livestock productivity index and the health status of animals due to impaired gut health, but natural alternatives such as supplemental *B. subtilis* in diets could ameliorate these effects (14). The probiotic effects of *B. subtilis* for different animals are highly strain-specific because of the different characteristics of the gastrointestinal environment (16). Therefore, it becomes imperative to discover which strains of *B. subtilis* are effective for broiler production concerning growth performance and health response.

Virginiamycin, a well-established AGP, has been widely used in the poultry industry and its growth-promoting effects have already been demonstrated in various studies (22, 23). In the present study, virginiamycin was taken as a positive control to evaluate the effects of two strains of *B. subtilis* A and B, and their combination (1:1) on the growth performance, serum immunity, and intestinal health of broiler chickens. The significant growth-promoting effects of virginiamycin in broiler chickens were validated again in this study. Numerous reports showed that broiler chickens direct-fed with *B. subtilis* showed enhanced growth performance than those fed a basal diet and comparably with the AGP group (14, 17, 18, 24, 25). However, in this study, although dietary *B. subtilis* significantly increased the ADG of broiler chickens, the improvement on both ADG and FCR was not comparable to AGP. The group fed the combination of BSA and BSB showed similar improvement on the ADG of broiler chickens relative to the AGP group. It may be due to the different strains and doses of *B. subtilis* used (18, 26). Remarkably, it is worthy to note that dietary *B. subtilis* in our study significantly decreased the mortality rate of broiler chickens relative to the AGP group. It is consistent with previous reports that the strain of *B. subtilis* reduced the pathology and improved the performance of broilers with necrotic enteritis induced by *Clostridium perfringens* challenge (27–29), and BSB alleviated diarrhea severity and systemic inflammation and improved gut health and growth performance of weaned pigs infected with enterotoxigenic *Escherichia coli* F18 (30). Therefore, it can be deduced that *B. subtilis* A and B probably decreased mortality and enhanced performance *via* enhanced gut health.

The immunoglobulin levels (IgG, IgA, and IgM) and lysozyme activity in the serum are important indicators to evaluate the non-specific immunity status of the animal (31, 32). Probiotics have been demonstrated to be beneficial immunomodulators of mammals at both phenotypic and molecular levels (33, 34). In the present study, dietary *B. subtilis* A and B significantly increased the contents of immunoglobulins IgG, IgA, and IgM in the serum to be comparable to AGP, and increased the lysozyme activity relatively higher than AGP. Also, for immune organ development, dietary *B. subtilis* A and B increased the weight of the thymus. The positive effects of these two strains of *B. subtilis* on immunity indices are consistent with previous reports that *B. subtilis* A and B significantly reduced the enteritis index of broiler chickens and the pathogenic bacteria-induced systemic inflammation of weaned pigs (30, 35). Other strains of *B. subtilis* were also reported to have auxo-actions on the immunity of broiler chickens, such as *B. subtilis* 1781, 747, DSM 29784, CPB 011, CPB 029, HP 1.6, and D 014 (14, 17, 36, 37). Therefore, the results of this study reveal that dietary *B. subtilis* could enhance the immunity of broiler chickens.

Intestinal barrier integrity is a prerequisite for the homeostasis of mucosal function to maximize the absorptive capacity and defense against chemical and microbial challenges (38). Gut commensals, referred to as probiotics, were discovered to reinforce intestinal health by impacting the intestinal barrier function (39, 40). Supplemental probiotics in diets can positively alter the intestinal micro-environment and promote early intestinal development (41, 42). In the present study, the dietary addition of *B. subtilis* significantly increased the relative weight and length of the small intestine relative to AGP, and the effects of dietary *B. subtilis* and AGP on intestinal morphology and barrier integrity were similar, both better than that of the control group. The results are consistent with the previous reports that birds fed BSA showed a high-efficient intestine with shallower crypt depth and higher villus height to crypt depth ratio, and pigs fed with BSB had enhanced gut health (11, 30, 35). Besides, the effects of the improvement on the epithelial barrier integrity and gut health were also reported with *C. butyricum, E. faecalis*, and other strains of *B. subtilis* (14, 17, 36, 37, 43). Therefore, we deduced that dietary *B. subtilis* A and B improved the intestinal barrier and enhanced the gut health of broiler chickens for better nutrient digestibility and utilization.

Gut microbes play vital roles in many aspects of animal health including immune, metabolic, and developmental traits (44, 45). Microbial fermentation results in the generation of SCFA, such as acetate, propionate, and butyrate, which can indirectly affect various physiological processes and may contribute to enhancing health or create a diseased state in the animal (46–48). SCFA can stimulate specific membrane-bound receptors to regulate aspects of intestinal motility, hormone secretion, maintenance of the epithelial barrier, and immune cell function (49). In the present study, dietary *B. subtilis* significantly increased the contents of lactic acids, succinic acid, and butyric acid in the ileum and cecum, and the contents of formic acid, isobutyric acid, and isovaleric acid in the cecum relative to the control and AGP groups. Butyrate is the main energy source for intestinal epithelial cells, propionate transferred to the liver regulates gluconeogenesis and satiety signaling, and other fatty acids produced also have been implicated directly in animal health outcomes (44). Therefore, it indicated that dietary *B. subtilis* A and B improved gut health of broiler chickens probably through increasing intestinal fatty acids production.

Probiotics and prebiotics are microbiota-management tools for improving host health (50). Probiotics have been used to prevent a wide range of diseases for decades, and studies have suggested positive effects of certain probiotics on gut microbiota balance (51). In the current study, dietary *B. subtilis* significantly increased the amount of *Lactobacillus* and *Bifidobacteria* in ileum and cecum and decreased the amount of *coliforms* and *Clostridium perfringens* in the cecum. *Lactic acid bacteria* and *Bifidobacteria* are well-known probiotics and are widely introduced into the food chain (52). Birds infected with pathogenic strains of *E. coli* show low performance because of high diarrhea and mortality rates (53). Acute necrotic enteritis caused by *Clostridium perfringens* infections results in severe losses in broiler production (37). The increased *Lactic acid bacteria* and *Bifidobacteria* and the reduced *Coliforms* and *Clostridium perfringens* induced by the dietary *B. subtilis* in the current study imply an improved intestinal micro-ecological balance. Therefore, these results showed that dietary *B. subtilis* A and B increased intestinal fatty acids production and gut health, probably through improving the balance of the intestinal microflora.

## Conclusion

Supplementation of BSA and BSB in broiler diets decreased mortality and enhanced growth performance. These positive effects could be accrued to efficient immune response capacity, intestinal microbial flora balance, the abundance of *Lactobacillus* and *Bifidobacteria*, and decreased *coliforms* and *Clostridium perfringens*, enhanced production of intestinal SCFA, and enhanced gut health. Also, the combination of two strains, BSA and BSB, synergistically enhanced intestinal morphology and integrity functions. Conspicuously, the results of the present study have provided strong evidence that supports supplementation of BSA and BSB in diets of broiler chickens as alternatives to synthetic antibiotics in the promotion of gut health and productivity index.

## Data Availability Statement

The original contributions presented in the study are included in the article/supplementary material, further inquiries can be directed to the corresponding authors.

## Ethics Statement

All experimental protocols were approved (AEC-CAAS-20191101) by the Animal Care and Use Committee of the Institute of Feed Research of the Chinese Academy of Agricultural Sciences.

## Author Contributions

Conceptualization, methodology, and project administration were performed by H-jZ and G-hQ. Animal experiment, chemical analysis, and data collection were conducted by KQ and C-lL. Original draft was written by KQ, C-lL, and H-jZ. Writing-review was finished by S-gW, JW, and JG. All authors have agreed to the final manuscript.

## Funding

This study was funded by the Shandong Key Science and Technology Innovation Program (2019JZZY010704), Beijing Innovation Consortium of Agriculture Research System (BAIC04-2021), Agricultural Science and Technology Innovation Program (ASTIP) of the Chinese Academy of Agricultural Sciences, and Evonik Degussa (China) Co., Ltd.

## Conflict of Interest

JG was employed by company Evonik (China) Co., Ltd. The authors declare that this study received funding from Evonik (China) Co., Ltd. The funder was not involved in the study design, collection, analysis, interpretation of data, the writing of this article or the decision to submit it for publication. The remaining authors declare that the research was conducted in the absence of any commercial or financial relationships that could be construed as a potential conflict of interest.

## Publisher's Note

All claims expressed in this article are solely those of the authors and do not necessarily represent those of their affiliated organizations, or those of the publisher, the editors and the reviewers. Any product that may be evaluated in this article, or claim that may be made by its manufacturer, is not guaranteed or endorsed by the publisher.
